# The *Caenorhabditis* Gli protein TRA-1 makes a transcriptional activator that promotes spermatogenesis

**DOI:** 10.1016/j.isci.2025.114108

**Published:** 2025-11-19

**Authors:** Yongquan Shen, Shin-Yi Lin, Yiqing Guo, Jibran Imtiaz, Kana Corley, Ronald E. Ellis

**Affiliations:** 1 Department of Cell and Molecular Biology, Rowan-Virtua School of Osteopathic Medicine, B303 Science Center, Stratford, NJ 08084, USA; 2 Department of Medicine, Renaissance School of Medicine, Stony Brook University, Stony Brook, NY 11794, USA

**Keywords:** Molecular biology, Chromosome organization, Biology of gender

## Abstract

Gli proteins are conserved transcription factors that regulate development. *Caenorhabditis* nematodes have a single Gli protein, TRA-1, which controls sex determination. Previously, it was thought that TRA-1 only produced a cleaved product that represses male genes. Here we show that full-length TRA-1 is an activator that promotes spermatogenesis. TRA-1 activator functions at the end of the sex-determination pathway in *Caenorhabditis briggsae.* The TRRAP homolog TRR-1 also acts at this point to promote spermatogenesis, so we propose that TRA-1 activator works with TRR-1 and the associated Tip60 HAT complex to turn on sperm genes. These results (1) reveal conservation between Gli activation in nematodes and humans, (2) show that Gli activation can occur without Hedgehog signaling, and (3) suggest an ancient role for Gli proteins in spermatogenesis. Finally, since a competition between TRA-1 activator and repressor helps determine germ cell fates, this regulatory flexibility might have facilitated the evolution of self-fertility.

## Highlights

• The C terminus of the Gli transcription factor TRA-1 controls germ cell fates
• Like other Gli proteins, the TRA-1 C terminus promotes transcription of target genes
• Nematodes do not require Hedgehog signaling to make TRA-1 activator
• TRA-1 activator requires the TRRAP protein TRR-1 to function

## Graphical abstract

**Figure:**
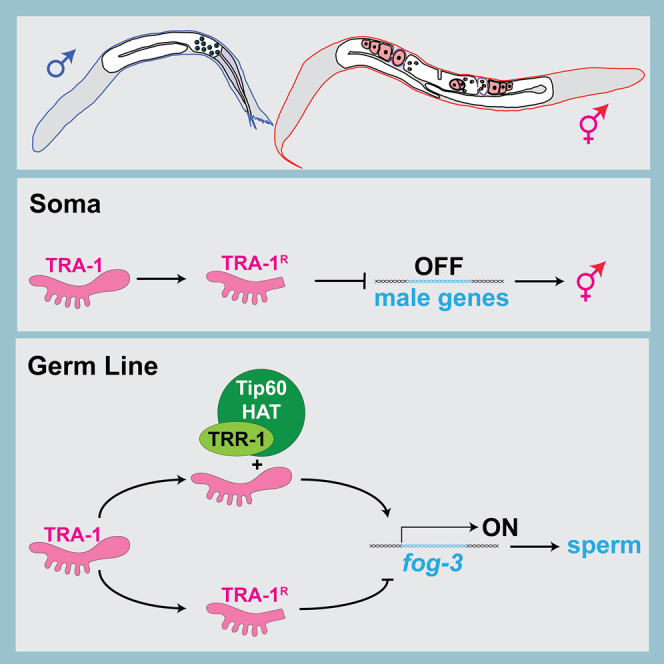

## Introduction

### Evolution of self-fertile hermaphrodites

**Figure 1:**
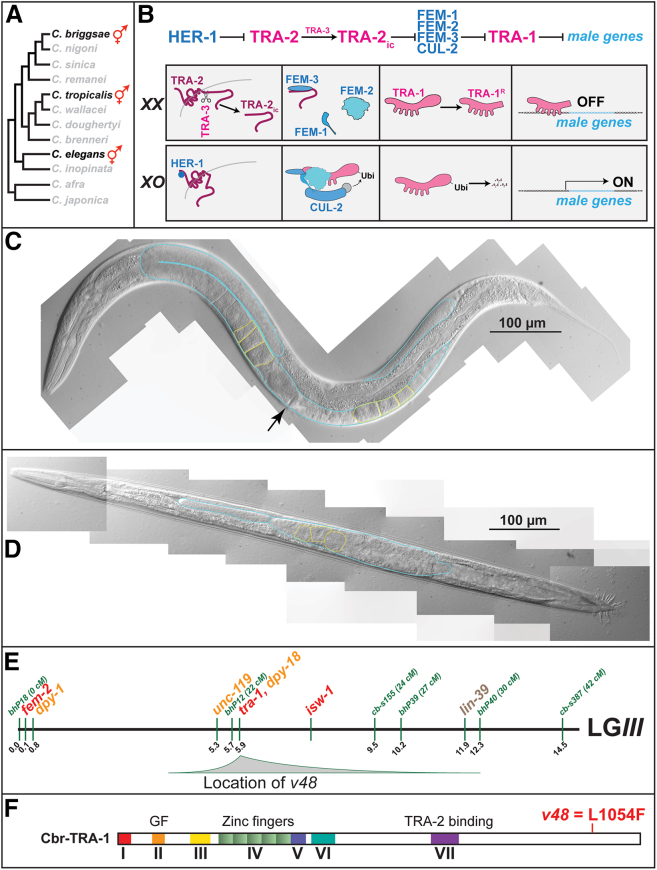
The *tra-1* mutation *v48* specifically alters germ cell fates in *C. briggsae* (A) Phylogeny of *Caenorhabditis* showing that ancestral species were male/female (shown in gray), but three species independently evolved *XX* animals that are self-fertile hermaphrodites. (B) (Top) Summary of core regulatory interactions that control sexual fates, with proteins that promote male fates in blue and those promoting female fates in red. (Bottom) In *XX* animals, the TRA-2 receptor is cleaved by TRA-3, releasing an intracellular fragment that binds FEM-3, preventing the FEM complex from working. This allows the TRA-1 transcription factor to be cleaved, forming a repressor of male genes. In *XO* animals, the male sex hormone HER-1 binds to and inactivates TRA-2, allowing the FEM complex to assemble with CUL-2 and ubiquitinate TRA-1, leading to its degradation. Hence male genes are expressed. (C) Photomicrograph of a *v48 XX* female raised at 20°C, with the gonad outlined in blue and some of the stacked oocytes in yellow; the vulva and empty uterus are indicated with an arrow. Anterior is left, ventral is down, and the scale bar represents 100 μm. (D) Photomicrograph of a *v48 XO* male raised at 25°C, with the gonad outlined in blue and oocytes in yellow. Anterior is left and the ventral side of the animal is shown. The scale bar represents 100 μm. (E) Genetic map of *C. briggsae* chromosome III, showing the location of *v48* as determined by two-factor mapping, with the shaded area representing the probability that *v48* is present at each position along the chromosome. (F) Diagram of *C. briggsae* TRA-1, showing the *v48* missense mutation.

Sexual development is critical for most animal species, since the proper formation of each sex underlies mating, reproduction, and the maintenance of genetic variation.^1^ Nematodes have undergone dramatic diversification in sex determination and mating systems during evolution.^2^^,^^3^ One of the most striking changes involves the formation of self-fertile hermaphrodites from female ancestors, a change that depends on altering the control of sex determination in germ cells.^4^ This transition to self-fertility has occurred three times during the evolution of the Elegans group of *Caenorhabditis* nematodes^5^ (Figure 1A).

### Sex determination in nematodes

In these nematodes, sex is controlled by a signal transduction pathway^6^ that responds to the ratio of *X* chromosomes to autosomes.^7^ The master regulator of sexual development is the last common gene in the pathway, *tra-1*.^8^^,^^9^ It encodes a Gli transcription factor related to *Drosophila* Cubitus interruptus and human Gli1, Gli2, and Gli3.^10^ The predominant form of TRA-1 is made by cleavage, which removes the C terminus^11^ (Figure 1B). The cleaved form promotes female development by repressing numerous male genes,^12^ including *fog-3* and *fog-1* in the germ line.^13^^,^^14^

### Cubitus interruptus and Gli proteins

Formation of TRA-1 repressor parallels the processing of other Gli proteins, since Cubitus interruptus is cleaved in fruit flies to make a repressor.^15^ Similarly, mouse Gli2 and Gli3 that lack their C termini act as repressors *in vitro,*^16^ and both genes can work as repressors *in vivo.*^17^

Beyond this similarity, it had seemed that TRA-1 differed significantly from Cubitus interruptus and mammalian Gli proteins. Most importantly, other Gli proteins act in response to hedgehog signals,^18^ but *Caenorhabditis* nematodes lack many of the key genes in this signal transduction pathway.^19^ In addition, although TRA-1 has extensive sequence homology to other Gli proteins in its five zinc fingers, there is no apparent homology elsewhere in the protein. Finally, studies with *Drosophila* revealed that Cubitus interruptus also *promotes* the transcription of some target genes.^20^ This activator function is carried out by the full-length (uncleaved) protein and works in conjunction with a small number of accessory proteins.^21^^,^^22^ Mammalian Gli1, Gli2, and Gli3 also produce full-length activators.

By contrast, there was no direct evidence that *Caenorhabditis elegans* TRA-1 could work as a transcriptional activator, although some epistasis experiments had raised that possibility.^23^ Here we demonstrate that full-length TRA-1 is indeed an activator, and that it promotes the transcription of sperm genes in nematodes. This activity is limited to the germ line but otherwise parallels activation by other full-length Gli proteins. These results indicate broad conservation of Gli protein function, even outside of the zinc-finger domain. Our observation that TRA-1 activator promotes the expression of *fog-3,* whereas TRA-1 repressor blocks it^13^ suggests a competition between activator and repressor in nematode germ cells. If so, this interaction could have played a major role in the evolution of self-fertility.

## Results

### Identification of a *Caenorhabditis briggsae tra-1* allele that feminizes the germ line

While screening *C. briggsae* for mutations that transformed *XX* hermaphrodites into true females,^24^ we recovered the mutation *v48.* At 20°C, homozygous *v48 XX* animals are females, having a hermaphrodite body but only producing oocytes (Figure 1C). However, the *XO* animals are normal males that produce sperm. Thus, a *v48* strain can be maintained as a mating population of males and females, much like *C. briggsae she-1* mutants.^24^ At higher temperatures, *v48* males sometimes switch to oogenesis later in life (Figure 1D), although they still form normal male bodies. Hence, *v48* has a classic Fog phenotype—it feminizes the germ line but not the soma.

Genetic mapping showed that *v48* is located on chromosome *III,* tightly linked to the *dpy-18* gene (Figure 1E). This region includes one known sex determination gene—*cbr*-*tra-1.* When we sequenced *tra-1* cDNA from *v48* animals, we found a single missense mutation near the C terminus of the protein (Figure 1F; Table S1). This region had not previously been defined by *tra-1* mutations, in either *C. elegans* or *C. briggsae.*

### A conserved domain of TRA-1 is required specifically for male germ cell fates

**Figure 2:**
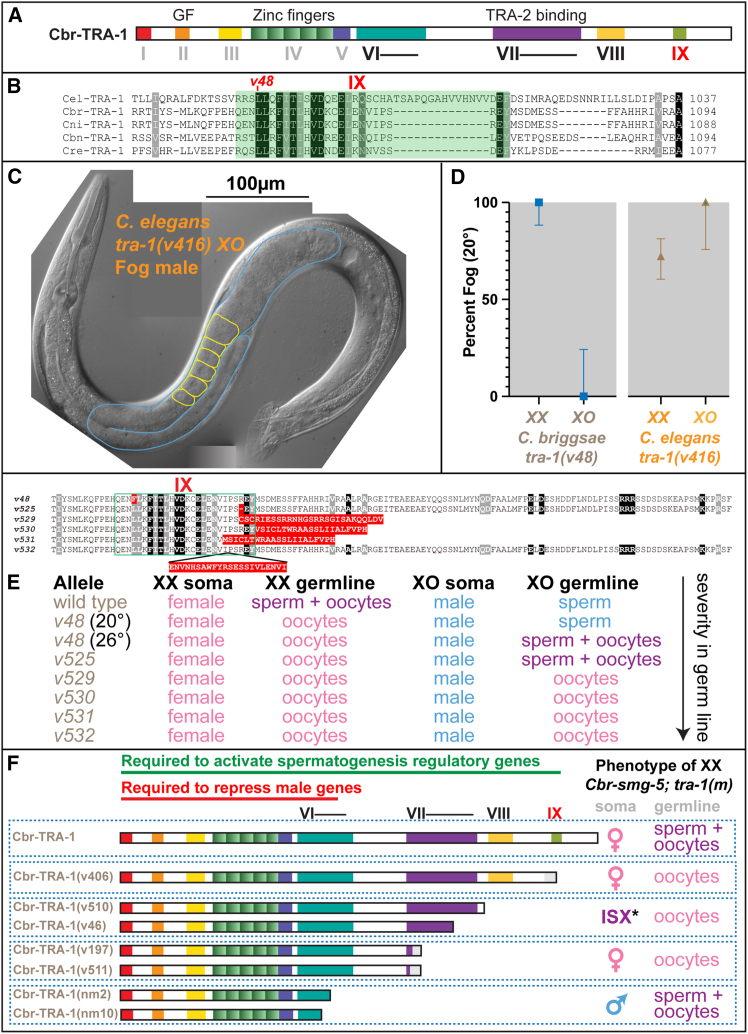
*v48* alters a conserved TRA-1 domain that promotes spermatogenesis (A) Diagram of *C. briggsae* TRA-1, showing key domains based on an alignment of the *C. briggsae, C. nigoni, C. elegans, C. remanei,* and *C. brenneri* proteins. Sequences are described in the STAR Methods, and a complete alignment is shown in Figure S3. (B) Partial alignment of TRA-1 sequences showing conserved domain IX and the location of the residue altered by *v48*. (C) Differential interference contrast photomicrograph of a *C. elegans tra-1(v416) XO* mutant*,* an allele causing the same amino acid change as *C. briggsae v48*. The scale bar represents 100 μm. (D) Graphs showing the differential effects of *v48* and *v416* on the two sexes at 20°C. Error bars show 95% confidence intervals calculated using the Wilson/Brown method, as implemented by GraphPad Prism. (E) Summary of the molecular changes and phenotypic effects of additional *C. briggsae* mutations made in domain IX by CRISPR-Cas9 gene editing. (F) Analysis of mutations that truncate *C. briggsae* TRA-1. All mutants were scored in a *smg-5* mutant background, to prevent nonsense-mediated decay. ∗The ISX animals had male gonads with oocytes, and partially masculinized tails.

To begin analyzing the *v48* mutation, we prepared an alignment of TRA-1 proteins from five species of *Caenorhabditis.* In addition to the domains described by de Bono and Hodgkin,^25^ we identified additional conserved sequences. First, regions VI and VII are both significantly larger than had been predicted from a two-way comparison of *C. elegans* and *C. briggsae* (Figure 2A). Second, two additional domains near the C terminus are also conserved, which we named VIII and IX (Figure 2A). The amino acid affected by *v48* is located in the newly discovered domain IX (Figures 2A and 2B) and is conserved in all five *Caenorhabditis* species.

To see if this identity in sequence reflected conservation in function, we used gene editing to make an equivalent mutation in *C. elegans.* This mutation, *Cel-tra-1(v416),* causes *XO* animals to produce oocytes instead of sperm (Figures 2C and 2D), but does not affect somatic male development. Moreover, many of the *XX* animals are females, although this trait is not fully penetrant (Figure 2D). Thus, this leucine-to-phenylalanine mutation has similar effects in both species—it causes animals to make oocytes instead of sperm but does not alter sexual development in the soma.

The tissue-specific effects of *Cbr-tra-1(v48)* and *Cel-tra-1(v416)* might reflect a unique role for domain IX in the germ line or could be due to a greater sensitivity of germ cells to the effects of these missense mutations. Thus, we used gene editing to produce additional *C. briggsae tra-1* mutations near the site of *v48* (Figure 2E; Table S1). These mutations have the same phenotype in *XX* animals as *v48*, transforming them into true females. Moreover, most also cause *XO* animals to produce only oocytes, without affecting the male body. Since these mutations range from a one-amino-acid deletion to a variety of frame-shifting alleles that cause early truncations, we infer that domain IX is only required in germ cells, and that it promotes spermatogenesis over oogenesis. By contrast, *tra-1* null alleles cause animals to (1) develop male bodies and (2) produce sperm predominantly or exclusively.^9^^,^^10^

One of these mutations caused an earlier truncation in the protein than the others and resulted in sterile *XX* animals rather than females (*v406,* Table S1; Figure 2F). We suspected that this frame-shifting allele triggered the nonsense-mediated decay system,^26^ resulting in the partial loss of *tra-1* transcripts. Thus, we built double mutants using an allele of the *C. briggsae smg-5* gene to prevent this loss.^27^ These *smg-5; tra-1* double mutants resulted in *XX* female animals and *XO* males that made oocytes. Hence, loss of domain IX causes germ cells to become oocytes but does not affect the soma.

To define the extent of TRA-1 that can be deleted without affecting the soma, we built additional *smg-5* double mutants with *tra-1* nonsense or frameshifting alleles located between the critical zinc finger domain and the C terminus (Figure 2F). Truncations in domain VI cause a strong loss of TRA-1 activity, favoring male fates in both the soma and germ line. By contrast, truncations in domain VII have the opposite effect, resulting in oogenesis rather than spermatogenesis. Moreover, one of these truncations is semi-dominant for feminization of the germ line despite having no effect on the soma. For example, 19.5% of *smg-5; tra-1(v197)/+ XX* animals developed as females, and only 80.5% as hermaphrodites (*n* = 185). Surprisingly, two truncations located more C-terminally not only feminize the germ line but partially masculinize somatic tissues (Figure 2F). We infer that the major function of the TRA-1 C terminus is to promote spermatogenesis. However, the weak semi-dominance in germ cells of a truncation in domain VII, along with the weak somatic effects caused by truncations in the VII-VIII region, suggests that these parts of the TRA-1 protein might be targets for additional regulatory interactions.

### The C terminus of TRA-1 controls transcriptional activation of *fog-1* and *fog-3*

**Figure 3:**
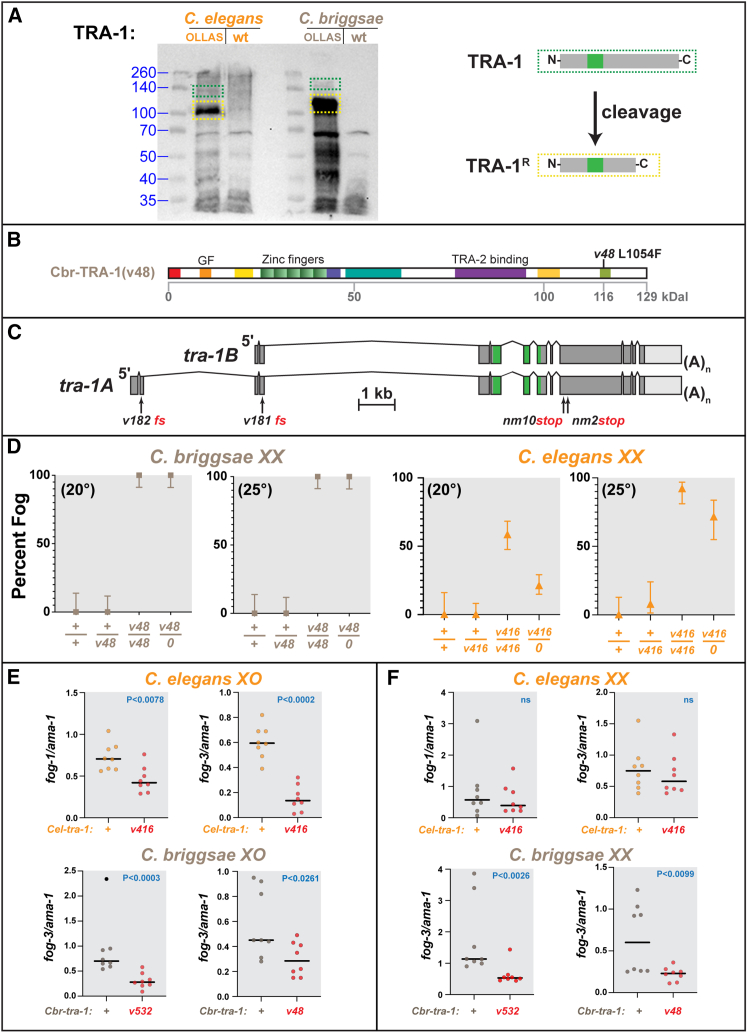
Full-length TRA-1 promotes expression of *fog-1* and *fog-3* (A) Western blot showing expression of TRA-1. For detection, TRA-1 was tagged with OLLAS near the N terminus, at position 49 in *C. elegans* and 129 in *C. briggsae* (STAR Methods). For each lane, 1,000 hand-picked worms were boiled in loading buffer. Dotted yellow lines indicate the cleaved repressor and dotted green lines the full-length protein. (B) Diagram of *C. briggsae* TRA-1 showing predicted molecular weights. Cleavage should occur near domain VIII. (C) Diagram of *C. briggsae tra-1* transcripts, showing four stop mutants. The scale bar represents 1 kb. The frameshift alleles *v181*^30^ and *v182* (this paper) remove most of the protein and transform *XX* animals into males. We used *v181* as the reference null allele because it alters both transcripts. Although *nm10* and *nm2* also masculinize *XX* animals,^31^*nm10* is weaker than *nm2,* so the correlation between their truncation points and activities is complex. (D) Gene dosage studies for *C. briggsae tra-1(v48)* and *C. elegans tra-1(v416).* Error bars show 95% confidence intervals calculated using the Wilson/Brown method, as implemented by GraphPad Prism. (E) Expression of the spermatogenesis genes *fog-1* and *fog-3* in *XO* animals, relative to the RNA polymerase gene *ama-1,* expressed in arbitrary units. Each point represents one animal. Averages are shown as bars, and the statistical significance of each comparison was calculated using the Mann-Whitney U test. (F) Expression of the spermatogenesis genes *fog-1* and *fog-3* in *XX* animals, as presented in (E).

Previous studies showed that a major activity of TRA-1 is repressing male genes like *fog-3*,^13^ *egl-1*,^28^ *mab-3*,^29^ and others.^12^ As with *Drosophila* Ci and human Gli proteins, repression is mediated by a cleaved form of the protein that lacks the C terminus.^11^ To confirm that *C. briggsae* TRA-1 also forms a cleaved repressor, we used gene editing to tag both *C. briggsae* and *C. elegans tra-1* with OLLAS near the N terminus (Table S1). Western analysis showed the predominant form of TRA-1 in both species is this cleaved repressor, which is roughly 100 kDa in size in *C. elegans*, and slightly larger in *C. briggsae* (Figure 3A). The full-length form is detectable in both species, but faint, as expected^11^ (Figure 3A). Molecular weight predictions imply that both domains VIII and IX are absent in the cleaved repressor (Figure 3B), so mutations like *v48* are unlikely to alter repressor activity.

Mutations that cause a loss of protein function are often recessive, whereas mutations that cause the loss of negative regulatory sites on a protein are often dominant. To see if the *v48* mutation caused a loss or gain of function, we used gene-dosage analysis. We began by confirming the *cbr-tra-1* null phenotype (Figure 3C). The *Cbr-tra-1(nm2)* mutation causes a strong transformation of *XX* animals from hermaphrodites to fertile males,^31^ but is located downstream of the coding region for the zinc fingers (shaded green), so it might retain some activity. Since *C. briggsae tra-1* makes two transcripts that differ at their N-termini,^25^ we also studied two early frameshifts made by gene editing—*cbr-tra-1(v181)* affects both the *tra-1A* and *B* transcripts,^30^ whereas *cbr-tra-1(v182)* affects only *tra-1A* (Figure 3C). Both mutations cause a strong transformation of *XX* animals into fertile males, much like *nm2*. Thus, we conclude that the *tra-1A* transcript is critical for function. Furthermore, we used *tra-1(v181)* as a reference null allele, since it affects both transcripts and truncates the protein before the zinc fingers. For *C. elegans* experiments we used *tra-1(e1099)* as our reference null allele,^8^^,^^9^^,^^10^ since it is a stop mutation that also truncates the protein before the zinc fingers.

To study gene dosage, we compared homozygous mutants with heterozygotes in *trans* to a wild-type or null allele (Figure 3D). The *C. briggsae tra-1(v48)* mutation is recessive and fully penetrant in *XX* animals, and *v48/null* animals resembled homozygotes, since they made oocytes instead of sperm but had normal hermaphrodite bodies. These results support the observation that *v48* only affects germ cells and are consistent with models in which it causes the loss of a TRA-1 function that promotes spermatogenesis.

The results for *C. elegans* were slightly more complex. Although *v416* was almost recessive, a few of the *tra-1(v416)/+ XX* animals were female at 25°, so that it is best described as weakly semi-dominant (Figure 3D). In addition, the homozygous *v416* animals were more often mutant than the *v416/null* controls. As with *C. briggsae,* we conclude that *C. elegans v416* specifically affects germ cells. However, our results are consistent with *v416* not only causing the loss of a TRA-1 function that promotes spermatogenesis but suggest that this mutant TRA-1 protein has weak dominant-negative effects. One possibility is that the mutant TRA-1 can titrate out co-factors needed for TRA-1 to promote the transcription of genes that favor spermatogenesis.

In *C. elegans*, *fog-1* and *fog-3* encode translational regulators that are directly responsible for germ cells initiating spermatogenesis rather than oogenesis.^32^ Both genes have several TRA-1-binding sites in their promoters,^13^^,^^14^ and these sites are targets for repression by TRA-1.^13^ Moreover, the functions of these genes are conserved in the *C. briggsae* germ line.^33^^,^^34^ Thus, a simple model is that *tra-1* regulates the levels of *fog-1* and *fog-3* transcripts to determine whether germ cells differentiate into sperm or oocytes.

If TRA-1 makes an activator that causes spermatogenesis by *promoting* the transcription of target genes like *fog-1* and *fog-3,* then both *Cbr-tra-1(48)* and *Cel-tra-1(v416)*, as well as similar mutations, should cause decreased expression of the *fog* genes. We used single worm RT-PCR^35^ to compare the expression of these genes with that of wild-type controls. First, we studied *XO* males during the 4^th^ larval stage (Figure 3E). In *C. elegans,* we saw significantly less expression of both *fog-1* and *fog-3,* relative to that of the RNA polymerase gene *ama-1.* When studying *C. briggsae* we focused on *fog-3,* which had shown the largest decrease in *C. elegans tra-1(v416)* males*.* Again, we saw a significant decrease in expression in the *tra-1* mutants, not only with the strong allele *v532,* but even with the missense mutation *v48,* which is temperature sensitive in *C. briggsae* males.

Next, we studied *XX* animals (Figure 3F). We observed significant decreases in *fog-3* expression for both *C. briggsae tra-1(v532)* and *tra-1(v48).* However, the decline in *fog-3* expression for *C. elegans tra-1(v416)* was small and not statistically significant. These results are consistent with the much stronger rate of transformation for *C. briggsae XX* mutants than for *C. elegans*.

Are the changes we saw in transcript levels sufficient to alter germ cell fates? In both *C. elegans*^14^^,^^36^ and *C. briggsae*,^34^ loss of a single copy of *fog-1* causes males to make oocytes. Thus, germ cell fates are very sensitive to small changes in *fog* gene expression. Here we observed decreases in both *fog-1* and *fog-3* levels, so their combined effects could well be decisive. We conclude that full-length TRA-1 has an activity separate from that of the cleaved repressor, that this activity is specific to germ cells, and that it promotes the expression of genes that regulate spermatogenesis.

### TRA-1 works with the TRRAP homolog TRR-1 to promote spermatogenesis

**Figure 4:**
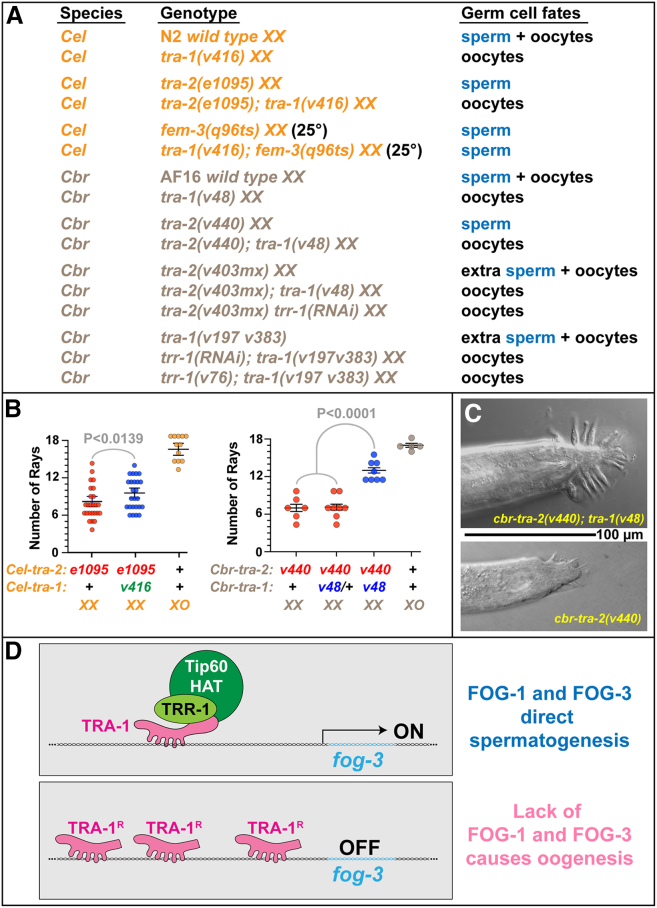
TRA-1 activator acts with TRR-1/TRRAP to regulate cell fates (A) Summary of double mutant phenotypes. *Cel* = *C. elegans, Cbr* = *C. briggsae*. (B) Number of rays visible on the tail. The *XO* males have 18 rays, although sometimes one or two are not visible because of how the animals were mounted on the slide. Each point represents one animal. Averages are shown as bars, bounded by the standard error for the mean. For comparisons, statistical significance was calculated using the Mann-Whitney U test. (C) Differential interference contrast photomicrographs of the male tail, ventral side up. The scale bar represents 100 μm. (D) Model for the regulation of germ cell fates by TRA-1 and the Tip60 HAT complex. In the top panel, full-length TRA-1 binds TRR-1 and Tip60 at its C terminus. Together, these proteins open up chromatin structure and promote *fog-*3 expression. In the bottom panel, the cleaved repressor binds the same target sites and prevents expression of *fog-3.* We infer that a competition between these isoforms could determine germ cell fates. Although both *fog-1* and *fog-3* are targets of TRA-1, only *fog-3* is shown for clarity.

To see if TRA-1 activator functions at the same step as the cleaved TRA-1 repressor, we analyzed double mutants (Figure 4). In *C. elegans,* TRA-1 activator works downstream of TRA-2 but upstream of FEM-3. Thus, it acts at the same point in the pathway as TRA-1 repressor. Furthermore, the repressor blocks expression of *fog-3*,^13^ and the activator promotes expression of *fog-3* (Figures 3E and 3F). Thus, both isoforms compete in the regulation of *fog-3,* like the Ci isoforms compete in the regulation of *dpp* in fruit flies.^37^

In *C. briggsae,* TRA-2 directly controls TRA-1 to determine germ cell fates.^35^ Our epistasis data indicate that TRA-1 activator acts downstream of TRA-2, so the simplest model is that TRA-2 binds TRA-1 to prevent the activator from promoting spermatogenesis.

We also analyzed double mutants to study the relationship between TRA-1 and the TRRAP protein TRR-1. First, the *tra-1(v48)* activator mutation is epistatic to *tra-2(v403mx),* and so are mutations that lower the function of *trr-1,* a homolog of TRRAP that is also needed for spermatogenesis^23^ (Figure 4A). Thus, TRA-1 activator and TRR-1 act at the same step in the sex-determination pathway. Furthermore, mutations in *trr-1* function are epistatic to a mutation in *tra-1* that causes hermaphrodites to make extra sperm (Figure 4A), whereas a complete loss of *tra-1* function is epistatic to *trr-1* mutations.^23^ Taken together, these results suggest that TRA-1 activator and TRR-1 act at the same point in the sex-determination pathway for germ cells. This conclusion makes sense, because TRA-1 is a transcription factor, and TRRAP a protein that aids transcription factors in forming regulatory complexes.^38^

Further genetic interactions involving *tra-1* and *tra-2* support this model. In both *C. elegans* and *C. briggsae, tra-2(null) XX* animals develop as males with severe defects in the formation of the tail.^8^^,^^31^ Previously, we showed that a loss of *trr-1* function partially suppresses these defects.^23^ Here we show that mutations in *tra-1* activator also suppress the tail defects observed in *tra-2 XX* mutants (Figures 4B and 4C). For unknown reasons, the suppression by both *tra-1* activator mutations and by *trr-1* mutations is much stronger in *C. briggsae* than in *C. elegans.* Taken together, these results suggest that the problem with tail development in *tra-2 XX* mutants is that TRA-1 activator is abnormally active in these somatic tissues and disrupts normal development. As a result, mutations that prevent the activator from functioning restore normal male tail development.

## Discussion

### Nematode TRA-1 produces a transcriptional activator

Gli proteins constitute an ancient and broadly conserved family of transcription factors. Although most functional studies have been carried out with fruit flies, vertebrates, and nematodes, they have been detected in many other animals, such as sea urchins.^39^ The founding Gli protein, *Drosophila* Ci, produces both a full-length activator and a cleaved repressor.^40^ Similarly, some vertebrate Gli proteins produce full-length activators and repressors that lack the C terminus.^41^ In nematodes, the predominant Gli isoform is a cleaved repressor^11^ that prevents the expression of male genes. For example, the repression of *fog-3* by TRA-1 results in oogenesis.^13^ However, some genetic studies have been consistent with *tra-1* producing additional products.^23^^,^^42^

Here we show that full-length TRA-1 *promotes* the expression of *fog-3,* favoring spermatogenesis*.* This conclusion is supported by mutations that affect the C terminus of TRA-1. These mutations have been isolated in two species, and in both they cause germ cells to differentiate as oocytes rather than as sperm. Since the domain they affect is removed during the formation of TRA-1 repressor, the full-length protein must promote the expression of *fog-1* and *fog-3*. Hence, TRA-1 resembles other Gli proteins far more than previously suspected and can be a valuable model for understanding Gli regulation and activity in mammals.

### Gli activation does not require hedgehog signaling

This aspect of TRA-1 expands our understanding of Gli regulation. The predominant model is that Hedgehog signaling causes Gli proteins to become translational activators, and that in its absence they form cleaved repressors.^15^^,^^43^ Although *Caenorhabditis* nematodes lack Hedgehog signaling,^19^ it had seemed that TRA-1 functioned only as a repressor, which was still consistent with this model.^12^ However, our demonstration that TRA-1 also produces an activator shows that Gli activation can occur independent of Hedgehog signaling. Perhaps non-Hedgehog control of Gli proteins also occurs in other animals. If so, analysis of Gli activation in *Caenorhabditis* might reveal conserved patterns of regulation.

For example, although nematodes lack Hedgehog signaling, the TRA-2 receptor is distantly related to Patched, a component of the Hedgehog pathway. This receptor can be cleaved to release an intracellular fragment that directly binds TRA-1. Our recent work shows that TRA-2/TRA-1 binding prevents spermatogenesis in *C. briggsae*,^35^ so it may oppose Gli activator function. Studies in human cells show that Patched can be cleaved to release an intracellular fragment that enters the nucleus, and that expression of this intracellular fragment modulates Gli reporter activity.^44^ Thus, control of TRA-1 by TRA-2 might help elucidate a new, direct path for regulation of mammalian Gli activators by Patched.

### TRA-1 and other GLI activators require Histone Acetyl Transferases

The full-length TRA-1 activator requires Histone Acetyl Transferase activity to function. Our previous work showed that nematode TRR-1 (the homolog of human TRRAP) is needed to promote spermatogenesis, and that TRR-1cannot do so in the absence of TRA-1.^23^ Furthermore, it showed that TRR-1 works with the Tip60 HAT complex in this role. Here we found that this relationship is reciprocal—a *tra-1* mutation that causes extra spermatogenesis in hermaphrodites requires TRR-1 for that effect. This interdependence suggests that TRA-1, TRR-1, and the Tip60 HAT complex not only work together to promote spermatogenesis but are likely to act at the same step in the regulatory process.

In fruit flies, the CREB-binding protein dCBP is a co-activator for the Gli protein Ci.^45^ Moreover, targeting CBP activity affects histone acetylation at Ci targets. CREB-binding proteins themselves have Histone Acetyl Transferase activity, and they can interact with the PCAF HAT complex.^46^ However, PCAF also regulates Gli1 through a separate ubiquitin ligase activity,^47^ so the nature of these interactions is unresolved. In nematodes, the critical tissue and promotors are known, so TRA-1 could provide an ideal model for studying Gli interaction with HAT complexes.

### TRA-1 activator specifically controls germ cell fates

In both *C. elegans* and *C. briggsae,* null alleles of *tra-1* affect all tissues, and transform *XX* animals into fertile males.^8^^,^^31^ By contrast, our analysis of *C. briggsae* shows that TRA-1 activator, indeed, the entire TRA-1 C terminus, specifically controls germ cell fates. Similarly, the *C. elegans tra-1(v416)* mutation suggests that TRA-1 activator does the same in that species. Moreover, several *smg-*sensitive alleles of *C. elegans tra-1* have been identified, and many of the suppressed *XX* animals are female rather than hermaphrodite.^48^ Since two of the alleles cause C-terminal truncations like those we characterized in *C. briggsae*^49^ (J. Hodgkin, Pers. Comm.), they support our conclusion that the C terminus of TRA-1 is needed to promote spermatogenesis. However, two other *smg-*sensitive alleles are missense mutations, which are not expected to be suppressed by blocking nonsense-mediated decay, so the *C. elegans* results should be interpreted with caution.

### Gli proteins could play a conserved role in spermatogenesis

Although TRA-1 regulates all aspects of nematode sex determination, neither *Drosophila* Ci nor human Gli1, Gli2, or Gli3 regulates sexual identity. Instead, these transcription factors control numerous other aspects of development, differentiation, and cell proliferation. However, our discovery that TRA-1 activator promotes spermatogenesis raises questions about the role other Gli proteins might play in germ cells. Gli1, Gli2, and Gli3 messages are all expressed in spermatogonia and spermatocytes.^50^ Moreover, Gli1 protein-DNA complexes have been purified from spermatocyte nuclei, and the overexpression of Gli1 blocks spermatogenesis in mice.^51^ In addition, a more distant member of the Gli family, the Gli-similar zinc finger protein GliS3,^52^ promotes spermatogenesis in mice.^53^ Thus, the role of TRA-1 in spermatogenesis might reflect an ancient role for Gli and Gli-like proteins in germline development.

### The bipotentiality of TRA-1 was critical for evolution of self-fertility in *C. briggsae*

Recently, we showed that an interaction between TRA-1 and the receptor TRA-2, which is cleaved to produce an intracellular fragment, lowers the amount of hermaphrodite spermatogenesis in *C. briggsae*.^35^ Although it was unclear why this would occur if TRA-1 only produced a cleaved repressor, our identification of TRA-1 activator suggests a simple model—full-length TRA-1 promotes spermatogenesis by promoting expression of *fog-1* and *fog-3* (Figure 4D), whereas the cleaved repressor prevents spermatogenesis by lowering their expression, as seen for *fog-3*.^13^ Thus, these two forms appear to compete to determine germ cell fates. In this model, the TRA-2 intracellular fragment binds TRA-1 to prevent the activator from promoting spermatogenesis (Figure 4D). Since the *fem-2* and *fem-3* genes play no detectable role in *C. briggsae* hermaphrodite spermatogenesis,^24^^,^^35^^,^^54^ self-fertility in this species appears to have evolved mainly by changes in the regulation of Gli activator in germ cells. These changes are likely to involve control of TRA-2 in *XX* animals by the newly evolved gene *she-1*.^24^

### Need for TRA-1 proteomics

Putting these results together, our discovery that nematode TRA-1 can function as an activator has already resolved many questions and suggests valuable lines for future work. For example, although TRA-1 is not required for male somatic development, it is expressed in many male neurons and altering TRA-1 levels has subtle effects on behavior.^55^ Analyzing the relative contributions of TRA-1 activator and repressor to these functions could elucidate how *tra-1* acts in the male nervous system. In addition, the existence of TRA-1 activator clarifies why *tra-2 XX* mutants make defective male tails, since these defects depend on Gli activator function. This result suggests that future research should focus not only on how TRA-1 activator and repressor control germ cell fates, but also on why TRA-1 activator does not normally alter somatic fates. As such, TRA-1 could be a good model for understanding how the dysregulation of Gli proteins leads to developmental defects.

However, dissecting the regulation of TRA-1 activator and repressor will be challenging. Although the repressor is readily detected, the full-length activator is barely detectable on western blots in *C. elegans*,^11^ and not at all in worms in which TRA-1 has been tagged with C-terminal GFP.^55^ Thus, the activator is likely to be present at very low levels in the germ line. Similar problems have been observed with TRA-2 in *C. elegans,* which is almost undetectable in germ cells, despite its clear role in controlling their fates.^56^ Hence future studies will require tagging and purifying TRA-1 and associated proteins from large quantities of nematodes, for mass spectrometric analyses of protein modification and cleavage. Fortunately, recent advances in gene editing make this feasible.

### Limitations of the study

These experiments involved genetic and molecular genetic tests of gene function and cannot reveal direct protein interactions. The data show that TRA-1 activator controls germ cell fates, but genetic mosaic analyses were not done to determine if it acts in the germ line to do this.

## Resource availability

### Lead contact

Further information and requests for resources and reagents should be directed to and will be fulfilled by the lead contact Ronald E Ellis (ellisre@rowan.edu).

### Materials availability

All strains are available upon request.

### Data and code availability

The data are presented in the manuscript. No code was generated for this work. Any additional information required to reanalyze the data reported in this paper is available from the lead contact upon request.

## Acknowledgments

Some strains were provided by the CGC, which is funded by 10.13039/100000002NIH Office of Research Infrastructure Programs (P40 OD010440). We thank 10.13039/100000002NIH Grants R01GM118836 and R01GM121688 for funding to R.E.E., and the 10.13039/100000048American Cancer Society for a Postdoctoral Award to support S.-Y.L. (126627-PF-15-228-01-DDC).

## Author contributions

Conceptualization, R.E.E., S.-Y.L., Y.G.; methodology, R.E.E.; investigation, Y.S., S.-Y.L., Y.G., J.I., K.C.; supervision, R.E.E.; validation, Y.S., J.I., R.E.E.; writing, R.E.E., Y.S., J.I.; funding acquisition, R.E.E., S.-Y.L.

## Declaration of interests

The authors declare no competing interests.

## STAR★Methods

### Key resources table

**Table:** 

REAGENT or RESOURCE	SOURCE	IDENTIFIER
**Antibodies**		

Rat Anti-OLLAS (L2) Antibody	Novus Biological	NBP1-06713; RRID: AB_1968650
HRP Goat Anti-Rabbit Secondary Antibody	Azure Biosystems	AC2118

**Chemicals, peptides, and recombinant proteins**		

Cas9 Nuclease protein	Horizon Discovery	CAS12206
Hot Start Taq Polymerase	New England Biolabs	M0495L

**Experimental models: Organisms/strains**		

*C. briggsae* wild type	Caenorhabditis Genetics Center	AF16
*C. elegans* wild type	Caenorhabditis Genetics Center	N2
*C. remanei*	Caenorhabditis Genetics Center	JU1184
*Cbr-smg-5(v246)*	Ellis lab	–
*Cbr-tra-2(v402mx)*	Ellis lab	–
*Cbr-tra-2(v403mx)*	Ellis lab	–
*Cbr-trr-1(v76)*	Ellis lab	–
*Cbr-tra-1(nm2)*	Eric Haag	–
*Cbr-tra-1(nm10)*	Eric Haag	–
*Cbr-tra-1(v181)*	Ellis lab	–
*Cbr-tra-1(v197v383)*	Ellis lab	–
*Cbr-dpy-18(mf104)*	Caenorhabditis Genetics Center	–
*Cel-tra-2(e1095)*	Caenorhabditis Genetics Center	–
*Cel-tra-1(e1099)*	Caenorhabditis Genetics Center	–
*Cel-fem-3(q96)*	Caenorhabditis Genetics Center	–

**Oligonucleotides**		

CGACAACCCACTCTCCATAA	This paper	RE1041 (Cbr-ama-1F)
GCCAATCGATGAAGATGTCAC	This paper	RE1042 (Cbr-ama-1R)
GCCAATGAAGTTCTCTAATATGAGACA	This paper	RE1012 (Cbr-fog-1F)
CAAAGTTCTGAAGTCCAGTCGATTC	This paper	RE1013 (Cbr-fog-1R)
TTCCACTCGCGTTGGAGAAG	This paper	YS01 (Cbr-fog-3F)
CGGATGTTGGCTTGAACGTG	This paper	YS02 (Cbr-fog-3R)
CCGACTCTCCACAAAATGTCA	This paper	RE1049 (Cel-ama-1F)
GGACGGCGCAGAGAGTATC	This paper	RE1050 (Cel-ama-1R)
GTTCAGATCGTTCCAGCGTC	This paper	RE1035 (Cel-fog-1F)
CAGAAGCTTCGTGGAATCCG	This paper	RE1036 (Cel-fog-1)
TTTGGCGCTGAACTTGGAAA	This paper	RE1033 (Cel-fog-3)
CATCGCAGTTCACATCTCCA	This paper	RE1034 (Cel-fog-3)

**Software and algorithms**		

Prism	GraphPad	–
AxioVision 4.8	Zeiss	–

### Experimental model and study participant details

All experiments were conducted with the nematodes *C. briggsae* and *C. elegans.*

#### Strains

*C. briggsae*: LGI : *smg-5(v246)*^27^; LGII : *tra-2(v402mx)*, *tra-2(v403mx)*,^35^ *trr-1(v76)*^23^; LGIII : *tra-1(nm2), tra-1(nm10)*,^31^ *tra-1(v181)*,^30^ *tra-1(v197v383)*,^35^ *dpy-18(mf104)*.^57^ *C. elegans*: LGII: *tra-2(e1095)*^8^; LGIII: *tra-1(e1099)*^8^; LGIV: *fem-3(q96)*.^58^ Mutations first described here are presented in Table S1.

### Method details

#### TRA-1 alignment

The *C. elegans, C. briggsae* and *C. nigoni* TRA-1 sequences have been described.^10^^,^^25^^,^^59^ By homology, we identified TRA-1 in the *C. brenneri*^60^ and *C. remanei*^61^ genomes, using data available on Wormbase (Figures S1 and S2). Because the *C. remanei* locus is near a partial duplication, we confirmed its structure by sequencing cDNA from strain JU1184 and aligning the exons with the PX506 sequence^61^ (Figure S2). Most of the *cre-tra-1* gene was amplified by RT-PCR, and the 3’ end by RACE.^62^ Complete alignments of all five TRA-1 proteins are shown in Figure S3.

#### Genetic mapping

From *dpy-18/ v48* mothers, we isolated 8 Dumpy progeny, none of which carried the *v48* mutation, and 16 non-Dumpy progeny, which included 9 *dpy-18/ v48* heterozygous hermaphrodites and 7 *v48* homozygous females.

#### Gene editing

We used TALENs and CRISPR/Cas9 as described.^27^^,^^59^

#### RNAi

We used RNA interference to knockdown *trr-1* expression in *C. briggsae tra-1(v197 v383)* and *tra-2(mx)* mutants, as described.^23^ The dsRNA concentration was 0.2 μg/μl.

#### Microscopy

Animal tails were observed using differential interference contrast microscopy. Images were captured using Zeiss AxioVision software.

#### Western blots

For each lane, 1000 young adults were picked into 10 μl of M9 solution, mixed with 10 μl of 4X SDS loading buffer, and boiled at 95-100°C for 5 minutes. The samples were then loaded onto an SDS-PAGE gel for separation, after which proteins were transferred onto a PVDF membrane. After a simple rinse with deionized water, the membrane was blocked with a 5% dry milk blocking buffer for one hour at room temperature. Afterwards, the membrane was incubated with primary antibody against OLLAS tag overnight at 4°C, then with secondary antibody for one hour at room temperature. Finally, it was developed using Bio-Rad Enhance chemiluminescent substrate for 5 minutes at room temperature, followed by three washes. The protein bands were detected by membrane exposure to X-ray film. The strains used for western blotting are Ollas-tagged *cbr-tra-1(v455), cel-tra-1(v472),* AF16 and N2.

#### OLLAS tags

CRISPR guide RNAs were designed according to Xu et al*.*^63^ We targeted the *C. briggsae* TRA-1 N-terminus using the gRNA: AGCGCAGCCCCTCCAAACAG, which lies between the gain-of-function and zinc finger domains. The single strand repair template was: ATAGCTTGGTTCCCGAAGCAGCGCAGCCCCTCCAATCTGGATTCGCGAACGAGCTTGGGCCCCGTCTTATGGGAAAGACAGTGGCTGAATCTTCGGAACCAACTGCAGCAAG. For *C. elegans,* the gRNA targeting TRA-1 was: TCGGAGGACAAACAACCTGG, located upstream of the gain-of-function domain, and the single strand repair template was: GCCAAGCAAATGGGTTCGGAGGACAAACAATCTGGATTCGCGAACGAGCTTGGGCCCCGTCTTATGGGAAAGCCTGGTGGTGGCGACGTGAAAACCGAAAATG. The protein Cas9, sgRNA, tracer RNA and single-strand oligos were mixed and microinjected into gonads of AF16 or N2 young adults. Animals with precise edits were identified by PCR screening and confirmed by DNA sequencing.

### Quantification and statistical analysis

Statistical analyses were performed using GraphPad prism.

For Figure 1E, the probability (in percentages) of observing 0/41 recombinants for a given map distance (MD) from *dpy-18* was calculated as: P = 100∗((100-MD)/100).^41^

For Figures 2D and 3D, error bars show 95% confidence intervals calculated using the Wilson/Brown method.

For Figures 3E, 3F, and 4B, averages are shown as bars, and the statistical significance of each comparison was calculated using the Mann-Whitney U test.
