# Supplementary material for: The Caenorhabditis Gli protein TRA-1 makes a transcriptional activator that promotes spermatogenesis
Source: iScience. 2025 Nov 19;28(12):114108. doi: 10.1016/j.isci.2025.114108 (PMC12719749; doi:10.1016/j.isci.2025.114108)
Supplement: Document S1. Figures S1–S3 and Table S1 [file mmc1.pdf]

iScience, Volume *28*

Supplemental information

The *Caenorhabditis* Gli protein TRA-1 makes a transcriptional activator that promotes spermatogenesis

Yongquan Shen, Shin-Yi Lin, Yiqing Guo, Jibran Imtiaz, Kana Corley, and Ronald E. Ellis

Supporting Information for:

The *Caenorhabditis* Gli protein TRA-1 makes a transcriptional activator that promotes spermatogenesis

Yongquan Shen
Shin-Yi Lin
Yiqing Guo
Jibran Imtaez
Kana Corley
Ronald E Ellis

This file includes:

Figure S1
Table S1

Genomic sequences taken from Cbre_Contig13: 1048309 - 1034640 (13,670 bp)

***Cbn-tra-1* cDNA (3450 bp)**

```
atgatggccccaagtaccgaggaccccgaaactgtcgtcgaggctcaacgtcgtggaagcttctccaagaagaagaacggatccggatggaccaacg
ctgagttggtcagtgaaaatggattggccaagtccatggattcgacaactggagagaacaagtcgaccatggaagttgatgagaaaaatgccataaa
cgatggaactcaagatctcacggccagaggcaacgcaaaagacgcgtcacctactcatggatcggccaccatcaagttcatcaactcatcagttcca
ccaactcatcaaactgagaccaatccattggagttgaatcctccagtcactcaaccagccaatggtgaggctactggtcaaccagctcaaccagcat
ctgagtctgagtcgaacggtgctccagctgagggagcagcagtagccactgctggatctgctgttccagttgtcaagttcaccaatcaaaccgcagc
taatggaagcactgtggccacttcagttggtcaaaacgttcgcctcactgttaacggcaagcgtgttgggcgtccaccaggcaccttcaaaagacca
ccgccaacaactacagccgctgctcatgctgatggtgatgttgatgttgaatcagtgagtgatctcatttgtcgctggaaggactgcatgcagaact
ttactactctcaaggcgcttgttgatcatgttcaagagaaacatgttcagtcaactgaacaagagcatcacgcatggcgttgtgaatgggagggatg
tgatcgtaatgagacattcaaagctctctacatgctcattgttcatgttcgtcgtcataccggagagaagccaaacaagtgcgagtatcccggatgt
ggaaaagaatacagtcgtcttgagaatctcaaaactcatcgccgcactcacaccggagagaaaccgtacaagtgcgagttcgctgattgcgagaaag
cgttcagcaacgcctcggatcgcgccaagcatcagaaccggactcattcaaatttgaaaccatatggatgccaaatcactggatgttctaaatcata
tactgatccatcatcacttcgtaaacacattaaagcagttcatggagatgatgagtacgagaaagcaaaaaagtctcgaccggcgaattactcaaat
cgccgccgtccagatatacgtatgcctccaccaactggagcactttctcatccatatctcgctccacctcatcactcaattcctcctacttctgcac
caatgcacactgttcagcagcaacaatttatcaatatggctttggttcaacaacatcaacgtgcacaattcatggcggcgactgccagtgtaccaat
gatggatccagctgcagctgcccaagtcacacaagcgcatcaggctcaaatgattcaaaatcagatgatgcaggcacaaatggcacaagcacaggca
atgcaacaacaacaagtcattcaagcacaggcaatgcaggctcaggcaatgcaagctcatgctatgcatcaagcacaggcattggctcttcagacga
acatcctcactgcacaaggactccttagcccattcccacagatgactccaatgcttccacaaagaccaccagcaaatgtgatggctcttcttcagca
tcaacatcaacaacagcagattcaacagagcactccacccactccaaccatgatgcagttcactccacatactccaattactccattgactccactt
actccaatgggaatgccaacagctgctccgattttcactatgccaactatgatggttgcagctccagttagcacggcgacgactgttatgccagctc
cagtcgttgctccagtgacgactcatgtctctccaactgctactacaactacatcaccacaattccgtcttcttcgtcaagaaatagaaacggcaga
tgctgacattgacccaatcagagtcgacggagatgatgaagatgatgacgcttcagaagatgaagaggaagaagcacgtgctcgtccagttggagca
gtaaccgctcaaagcaatggaggaggatctgatgatcacagtgcatcgggagcctcttcctctcgttcaagtgcttcaagtggcagtggaacattgg
aaattgctccagtacagaatggaaaccgtagcagcagcactggagatcgtggaatgagaagcttcctgattgctgatattcttcagctcgcttgtga
cttcaagaacgatcgtcttctcactgatgcgttggatcttgccgtctttggaactcctgatctcaaaactcttcacaagatctatcaactgtatgaa
cgtatttcccatgaattcgtttcaccaagccgtcgtttcattagctggcaagaagtcaatcttctccatcaatactatcatcatccacgtttcaatc
gtgctggctatcatgaatctccattgacacgtactcgcgagcagctcttctggcgtgttgtcaactgggtcaacttgaacagtactcgtcaaatcca
gccagtttcattagatagagatgatgatgagggatactttgatgagatggcttcaatgatgaatcatgctcatgtcaatgtaaacaaccacttcgca
gacgagatggttgatgatggatttgatgtcagtgatattgaagatgatgatgaagatgttgttggattcggaggagatattggagttgctattcatc
gtcgccgtcgtcaagttcgtcatcaagcactgaaacaagcatacattgatttcgatgccgatgaaggattcggaaatgccgatggtggatttggagc
agaaggcgacggagatggacacagaaacggtgatccatacttaaatgatgaagctcgtacatgtcgtctcaacatagaaatgaacgttgaggaagag
aaatgtgcatcaatggaggaattcctgaagaaacatgaagaggctcgtgccagatacgatgcctacaaggagaagcaagtagaattaataatgaatc
gttcaattgatgataaagaactcaagatgccagttggaaatggcattccttgtgatccagttccagctgccgagtcatcaacttccagccgtcgttc
aagtgttagcagaatgctcgttgaggaaccagcaactcgttcaactcttcttcgtttcgtcacaatccacgttgacagagaggaaatccggaatcaa
attccttcgttggagtatgtggagactccacaatctgaagaggatagcctggaagcacagcatcgtattgctgttgaagcattagctggattctccc
gaatgacactcaatgatcgacaatcagagacgctcaccccctgaagatgagccagtcgccgtcaatccagatgacgcgttcaatgattcccgtcgtcg
tcattatacttctcgtgacaacaatgttccagagaagaaatctcgtgatctctga
```

Cbn TRA-1 (1149 aa)

```
MMAPSTEDPETVVEAQRRGSFSKKKNGSGWTNAELVSENGLAKSMDSTTGENKSTMEVDEKNAINDGTQDLTARGNAKDASPTHGSATIKFINSSVP
PTHQTETNPLELNPPVTQPANGEATGQPAQPASESESNGAPAEGAAVATAGSAVPVVKFTNQTAANGSTVATSVGQNVRLTVNGKRVGRPPGTFKRP
PPTTTAAAHADGDVDVESVSDLICRWKDCMQNFTTLKALVDHVQEKHVQSTEQEHHAWRCEWEGCDRNETFKALYMLIVHVRRHTGEKPNKCEYPGC
GKEYSRLENLKTHRRTHTGEKPYKCEFADCEKAFSNASDRAKHQNRTHSNLKPYGCQITGCSKSYTDPSSLRKHIKAVHGDDEYEKAKKSRPANYSN
RRRPDIRMPPPTGALSHPYLAPPHHSIPPTSAPMHTVQQQQFINMALVQQHQRAQFMAATASVPMMDPAAAAQVTQAHQAQMIQNQMMQAQMAQAQA
MQQQQVIQAQAMQAQAMQAHAMHQAQALALQTNILTAQGLLSPFPQMTPMLPQRPPANVMALLQHQHQQQQIQQSTPPTPTMMQFTPHTPITPLTPL
TPMGMPTAAPIFTMPTMMVAAPVSTATTVMPAPVVAPVTTHVSPTATTTTSPQFRLLRQEIETADADIDPIRVDGDDEDDDASEDEEEEARARPVGA
VTAQSNGGGSDDHSASGASSSRSSASSGSGTLEIAPVQNGNRSSSTGDRGMRSFLIADILQLACDFKNDRLLTDALDLAVFGTPDLKTLHKIYQLYE
RISHEFVSPSRRFISWQEVNLLHQYYHHPRFNRAGYHESPLTRTREQLFWRVVNWVNLNSTRQIQPVSLDRDDDEGYFDEMASMMNHAHVNVNNHFA
DEMVDDGFDVSDIEDDDEDVVGFGGDIGVAIHRRRRQVRHQALKQAYIDFDADEGFGNADGGFGAEGDGDGHRNGDPYLNDEARTCRLNIEMNVEEE
KCASMEEFLKKHEEARARYDAYKEKQVELIMNRSIDDKELKMPVGNGIPCDPVPAAESSTSSRRSSVSRMLVEEPATRSTLLRFVTIHVDREEIRNQ
IPSLEYVETPQSEEDSLEAQHRIAVEALAGFSRMTLNDRQSETLTPEDEPVAVNPDDAFNDSRRRHYTSRDNNVPEKKSRDL
```

Figure S1. *C. brenneri* TRA-1. Prediction of TRA-1 protein sequence for *C. brenneri*. The genomic sequences were provided by Wormbase, and the predicted gene structure and cDNA sequence was based on homology to *C. elegans* and *C. briggsae* TRA-1 sequences.

Genomic sequences taken from Cre PX506 III: 9548128-9561637 (13,510 letters)

Cre-tra-1 cDNA (3436 bp)

```
atgatggccccaagtactgaggaccccgaaactgttgttgatgctcaacgtcgtggcagcttctcaaagaagaaaaatggaaatggatggagtttgg
gaaatgagaatgatttggctaaaaacatggaaccactcacggattgtaagactgcgatggatgttgatgatcagggttccaaatcagatggttctgg
tagccctctcaaagagaaaagcccatcacttggatcagcaactgccaatttcattcgttcatccgttgcaccatcccatcaaactgctcaaaatcca
ttggaacttcgtccaccatcaccagtgaaccaatccgcagacactgttactcaattagttggtacagctcaagcgattcaatcgtctcaaccagttg
tcgaatcatctgctcccgcgactgccccatctgaagatcaagcaattgcagcgtcatcttctaatccagtttctgtggttcccgttgtcaaattcac
aaatcaaacagctccaaatgggagtactgtagccacctcagttggtcaaaacgtccgtcttaccgtcaacggaaaaagagtgggacgtccaccagga
acattcaaaagagcaccaaaccacatcgcaagctcgaataataacggagacgatgttgatgtagagacggcaaatgatctgacttgtcgatggaagg
attgtatgatgaagttctcgactctcaagggcttggttgatcatgttcaagaaaagcacgttcaatcaactgaacaagaacaccatgcatggcgttg
tgaatgggaaggatgtgatcggaatgagacgttcaaagcactctatatgctcattgtgcatgttcgtcgtcacacgggagagaagccgaacaagtgt
gaatatcccgggtgtggaaaagaatacagtcgtcttgaaaatctcaaaacgcatcgaagaactcacaccggagagaaaccctacaaatgtgaattcg
ctgattgtgaaaaagcgttcagtaatgcttctgatcgcgccaaacatcagaatcgtactcattccaacttgaaaccatatgcatgtcaaatcatcgg
ctgccagaagtcgtacactgatccatcttcacttcgaaagcacatcaaagctgttcatggtgatgaggaatatgagaaagcaaaaaagtcacgtcca
ccaaattattcaaatcgtagacgtcctgatcctcgaatggctccaccaactggatccctctctcatccatatctctcaacacctcattccatgactt
caaatgcagttccagttcaccagaacaactttatcaatttggcattggctcaacatcatcatcgggctcaacttatggcgagcaatggagcgttgat
ggatccaactacggcggttcaagtatctcaagctcatcaggctcatatgatgcaatctcatatgatgcagcaagctcatgttaatgctgtggctgct
gctcaaattcaagctcaacatcaagcaatgcaggttcaggcaatgcaacaggctcaagtaatgcaacatgcacaagcaatgcaacaagctcaagcca
tggttctccagaataacatcctgtcggcacagggacttctcaatccattcacaccaatttctccattacttccaccaagaccaacgagtatgatggc
tcttattcatcatcaacaacaacaacaacagcaacaacaaggacaacaattgactccgcccactccaacaatgatgcagttgactccacatactcca
attactccaatcactccaatcactccgatgggatcgaatggtccaatgttcactatgcctaacctagtggttcagcctcctattcgggaaccaattc
gagttgatactgtcgctccgcccactggtgttcatccgacgaatcaatcagcacgaccaaatttcccatctatccctcaacagattcatttgcataa
cgcaaatgtcgcaacggctgaagaagatgatgaaatgtcagaagatgatgaagaatctcttcaacctgctcaggcaatccctgcttcccgtcccaga
gacggatcagatgacgggaacagtggttccggtgctggttcttctcgttctagtgtttcgagtggaagtggaactttggaaatggctggaagagcaa
ctcagagtgaaagcagaagcagcggatctggtgaacgtggaatgagaagtttcttaattgctgatatccttcaactcgccagtgaattcggaaatga
tcgtatcatttcagacgctctcaatctctccatcttcgaaactcgtgatatcaacaccgtctggcatatatatagtctattcgacaatgcttgctcc
cgaatggatcatttgaaacaggaaaacgtcttccaacttggcaagaagttcgagttcttcattcttattaccatagtccacattacaatcgtaatc
ttttccacgattccccagctgcccgtactcgtgaaactcttttctggcgcgcgatcaactctgcaaatagccgcagagaacatcaaattcaatcgat
ttcacttaactctgaagttgatgaaggatttgaagaatatgctcttcgagcagctcgtgacggtagacgtgctacttcgaataatcaaagtgctgct
gttagagttggagctcaagaatctgaagatgaagatgatggttttggtgattcagatgatgacctcccaggtctcggattgggaggtttggatgtta
ttgttcgaagaagacgtcgaattgtccgtcgtcaagctttgaaacaagcttatatggagattgatgaaagcaataccgacaaccagtttggagttgt
tggaggaggattcggtggcgaaaacaatgaaaatggagacgaccgaagctatgatagctttgttcatacacctcgaatcgatgattcagatatcaat
tttataaagttattcaagagagccgagtctgatcttgctgagaaagcagcaaagttgccaaaaattactgaaatggttccaatagatcaagttcaga
catctcagcaaatccaaccagaaccaatcaaccctccagctgcctgccgatcgagcacaactcctttcagcgttcacagattgttggtcgaagaacc
ggaatttcgtcaaagtcttctccgtttcgtaacaattcatgttgacaacgatgagattaaaaataacgtctcatcggatgagttctacaaattgcca
tcagacgagagacggatgattgaagaagctatgtcagctatggatggtatggagtcggctgaagctgcccagaatcaagctgtgattgaagaaccac
aagaatcgctgttcgcagaagaccaagaatcatcatctaatgttccaagtgactattccattccttccaatccacgtcgtcgctccagtgattcgga
gcattcagaagtcgtcccagagaagaaacaacgcaaacattaa
```

Cre TRA-1 (1145 aa)

```
MMAPSTEDPETVVDAQRRGSFSKKKNGNGWSLGNENDLAKNMEPLTDCKTAMDVDDQGSKSDGSGSPLKEKSPSLGSATANFIRSSVAPSHQTAQNP
LELRPPSPVNQSADTVTQLVGTAQAIQSSQPVVESSAPATAPSEDQAIAASSSNPVSVVPVVKFTNQTAPNGSTVATSVGQNVRLTVNGKRVGRPPG
TFKRAPNHIASSNNNGDDVDVETANDLTCRWKDCMMKFSTLKGLVDHVQEKHVQSTEQEHHAWRCEWEGCDRNETFKALYMLIVHVRRHTGEKPNKC
EYPGCGKEYSRLENLKTHRRTHTGEKPYKCEFADCEKAFSNASDRAKHQNRTHSNLKPYACQIIGCQKSYTDPSSLRKHIKAVHGDEEYEKAKKSRP
PNYSNRRRPDPRMAPPTGSLSHPYLSTPHSMTSNAVPVHQNNFINLALAQHHHRAQLMASNGALMDPTTAVQVSQAHQAHMMQSHMMQQAHVNAVAA
AQIQAQHQAMQVQAMQQAQVMQHAQAMQQAQAMVLQNNILSAQGLLNPFTPISPLLPPRPTSMMALIHHQQQQQQQQGQQLTPPTPTMMQLTPHTP
ITPITPITPMGSNGPMFTMPNLVVQPPIREPIRVDTVAPPTGVHPTNQSARPNFPSIPQQIHLHNANVATAEEDDEMSEDDEESLQPAQAIPASRPR
DGSDDGNSGSGAGSSRSSVSSGSGTLEMAGRATQSESRSSGSGERGMRSFLIADILQLASEFGNDRIISDALNLSIFETRDINTVWHIYSLFDNACS
RMDHFETGKRLPTWQEVRVLHSYYHSPHYNRNLFHDSPAARTRETLFWRAINSANSRREHQIQSISLNSEVDEGFEEYALRAARDGRRATSNNQSAA
VRVGAQESEDEDDGFGDSDDDLPGLGLGGLDVIVRRRRRIVRRQALKQAYMEIDESNTDNQFGVVGGGFGGENNENGDDRSYDSFVHTPRIDDSDIN
FIKLFKRAESDLAEKAAKLPKITEMVPIDQVQTSQQIQPEPINPPAACRSSTTPFSVHRLLVEEPEFRQSLLRFVTIHVDNDEIKNNVSSDEFYKLP
SDERRMIEEAMSAMDGMESAEAAQNQAVIEEPQESLFAEDQESSSNVPSDYSIPSNPRRRSSDSEHSEVVPEKKQRKH
```

Figure S2. ***C. remanei*** **TRA-1.** Prediction of TRA-1 protein sequence for *C. remanei.* . The genomic sequences were provided by Wormbase, and the predicted gene structure and cDNA sequence were confirmed as described in the Methods.

```
          Domain I                                                                                   Domain II
Cel-TRA-1 MMAPSTEDPDTVVEAQRRGSFSKKKNANGWNKVELVDQC--AKQMGSEDKQP---GGGDVKTEN-------D-----PSKNG---LGSATSNFIQSSVPP 80
Cbr-TRA-1 MMAPSTEDPETVVEAQRRGSFS-KKNGNGWTKPGLANGSE-SKHMEPVTGTNALMAAIDADDQETKLK---D-----PLEKEASTYKSATDRFFQSSHPP 90
Cni-TRA-1 MMAPSTEDPETVVEAQRRGSFS-KKNGNGWTKPGLANGSE-SKHMEPVTGTNALMAAIDADDQETKLK---D-----PLEKEASTYKSATDRFLQSSHPP 90
Cbn-TRA-1 MMAPSTEDPETVVEAQRRGSFSKKKNGSGWTNAELVSENGLAKSMDSTTGENK--STMEVDEKNAINDGTQDLTARGNAKDASPTHGSATIKFINSSVPP 98
Cre-TRA-1 MMAPSTEDPETVVDAQRRGSFSKKKNGNGWS---LGNENDLAKNMEPLTDCK---TAMDVDDQGSKSDGSGS-----PLKEKSPSLGSATANFIRSSVAP 89

                                                                                   Domain III
Cel-TRA-1 SHQTLSNPLQLSPPAE-------ASVAQQSGASQVFPTFQAALGASSDELL--QPNATSSSTSSSASTSSIVPVVKFTNQTAPNGSTVATSVGQNVRLTI 171
Cbr-TRA-1 SHQTVENPIKLEPPTSSGVTQNGSPVTNSLVPEAA-QPLQTVAESSEPTAASAEPTDASSAESSTITVTPVVPVVKFTNQTTPNGSTVATSVGQNVRLTI 189
Cni-TRA-1 SHQTVENPIRLEPPTSSGVTQNGSLVTNSLVPEAA-QPLQTVAESSEPPAASAEPTDASTAEPSTISATPVVPVVKFTNQTTPNGSTVATSVGQNVRLTI 189
Cbn-TRA-1 THQTETNPLELNPP-----------VTQPANGEATGQPAQPASESESNG-A---PAEGAAVATAG----SAVPVVKFTNQTAANGSTVATSVGQNVRLTV 179
Cre-TRA-1 SHQTAQNPLELRPPSP--VNQSADTVTQLVGTAQAIQSSQPVVESSAPATA---PSEDQAIAASSSNPVSVVPVVKFTNQTAPNGSTVATSVGQNVRLTV 184

                                                          Domain IV Zn Fingers
Cel-TRA-1 NGKRVGRPPGTFKRPQNNAANSSNSGNDSDMMGDHDLTCRWKSCNSSFQTLKALVDHVQESHVQSTEQEHHAWRCEWEGCDRNETFKALYMLIVHVRRHT 271
Cbr-TRA-1 NGKRVGRPPGTFKRPPNNPTTNTSHGEETDSEASTDLICRWKDCMLKFANLKGLVEHVQEKHVQSTEQEHHAWRCEWEGCDRNETFKALYMLIVHVRRHT 289
Cni-TRA-1 NGKRVGRPPGTFKRPPNNPTTNTSHGEETDSEASTDLICRWKDCMLKFANLKGLVEHVQEKHVQSTEQEHHAWRCEWEGCDRNETFKALYMLIVHVRRHT 289
Cbn-TRA-1 NGKRVGRPPGTFKRPPPTTTAAAHADGDVDVESVSDLICRWKDCMQNFTTLKALVDHVQEKHVQSTEQEHHAWRCEWEGCDRNETFKALYMLIVHVRRHT 279
Cre-TRA-1 NGKRVGRPPGTFKRAPNHIASSNNNGDDVDVETANDLTCRWKDCMMKFSTLKGLVEHVQEKHVQSTEQEHHAWRCEWEGCDRNETFKALYMLIVHVRRHT 284

Cel-TRA-1 GEKPNKCEYPGCGKEYSRLENLKTHRRTHTGEKPYKCEFADCEKAFSNASDRAKHQNRTHSNLKPYSCQIPQCTKSYTDPSSLRKHIKAVHGDDEYEKAK 371
Cbr-TRA-1 GEKPNKCEYPGCGKEYSRLENLKTHRRTHTGEKPYKCEFSDCEKAFSNASDRAKHQNRTHSNLKPYSCQIGGCQKSYTDPSSLRKHIKAVHGDEEYEKAK 389
Cni-TRA-1 GEKPNKCEYPGCGKEYSRLENLKTHRRTHTGEKPYKCEFSDCEKAFSNASDRAKHQNRTHSNLKPYSCQIVGCQKSYTDPSSLRKHIKAVHGDEEYEKAK 389
Cbn-TRA-1 GEKPNKCEYPGCGKEYSRLENLKTHRRTHTGEKPYKCEFADCEKAFSNASDRAKHQNRTHSNLKPYGCQITGCSKSYTDPSSLRKHIKAVHGDDEYEKAK 379
Cre-TRA-1 GEKPNKCEYPGCGKEYSRLENLKTHRRTHTGEKPYKCEFADCEKAFSNASDRAKHQNRTHSNLKPYACQIIGCQKSYTDPSSLRKHIKAVHGDEEYEKAK 384

              Domain V
Cel-TRA-1 KSRPANYSNRRRPDHRLAPPTGAMSHPYLATPNSGASVVAHSSVH---QQNFINMALAQHHHNAQRAQQLMAATGNVMPMMDPAS--AAAAAQAQAHHQA 466
Cbr-TRA-1 KSRPPNYSNRRRPDHRMPPPTSAMSHPYLAPP-HVLPPVAAPGVH---QQNFINLALAQHHHNQR--AQFIANNAA---LMDPNGSGAAQAAAAAQAAQA 480
Cni-TRA-1 KSRPPNYSNRRRPDHRMPPPTSAMSHPYLAPP-HVLPPVAAPGVH---QQNFINLALAQHHHNQR--AQFIANNAA---LMDPNG--SAQAAAAAQVAQA 478
Cbn-TRA-1 KSRPANYSNRRRPDIRMPPPTGALSHPYLAPPHHSIPPTSAP-MHTVQQQQFINMALVQQHQ--R--AQFMAATASV-PMMDPAA--AAQVTQAHQAQMI 471
Cre-TRA-1 KSRPPNYSNRRRPDPRMAPPTGSLSHPYLSTP-HSMTSNAVP-VH---QNNFINLALAQHHH--R--AQLMASNGA---LMDPTT--AVQVSQAHQAHMM 470

                                          Domain VI
Cel-TRA-1 Q---------AQMLQTHMMQQAQIQAAAQMQAQVQHQAAMQAHAMQQAQ-MVLQNNLLGAQSLLSPFS---PLLPPSRAPNVMAMLQ------------ 540
Cbr-TRA-1 QQ--------AQMYQAQAMQQHQF--AAQMQQQAVMQVHMQQAALQQAQVAIIQNNLLSTQGIMNQFPMMSPLLTPRAPGNVMSLLQQQQV--------- 561
Cni-TRA-1 QQ--------AQMYQAQAMQQHQF--AAQM----QMQAHMQQAALQQASAAIIQNNLLSTQGIMNQFPMMSPLLTPRAPGNMMSLLQQQQV--------- 555
Cbn-TRA-1 QNQMMQ----AQMAQAQAMQQQQ---VIQAQAM-QAQAMQ-AHAMHQAQALALQTNILTAQGLLSPFPQMTPMLPQRPPANVMALLQHQHQQQQIQQS-- 560
Cre-TRA-1 QSHMMQQAHVNAVAAAQIQAQHQ---AMQVQAMQQAQVMQHAQAMQQAQAMVLQNNILSAQGLLNPFTPISPLLPPR-PTSMMALIHHQQQQQQQQQGQQ 566

Cel-TRA-1 -TPPTPT-------------------------SVAPMFDI--MTSRAPMA-----------PVVSAPTAPAPLVPAPVPAS-----------PVFDELRE 590
Cbr-TRA-1 -TPPTPTMMQLTPHTPITPITPITPITPLTPMG-ATTGPMFSMPNIMMTTPVRMDIPTSVPAAVPVAPVAPV-APVAP-AAPVAPPASSLAEATEPRFRLLRQ 657
Cni-TRA-1 -TPPTPTMMQLTPHTPITPITPITPITPLTPMG-ATTGQMFSMPNIMMTTPVRMDIPTSVPAAVPVVPVAPV-APVAP-AAPVA-PATSLAEAAEPRFRLLRQ 650
Cbn-TRA-1 -TPPTPTMMQFTPH---TPITPLTPLTPMGMPTAAPIFTMPTMMVAAPVS--------TATTVMPAPVV-APVTTHVSPTA------TTTSPQFRLLRQ 641
Cre-TRA-1 LTPPTPTMMQLTPH---TPITPITPITPMG--SNGPMFTMPNLVVQPPIR-----------EPIRVDTV-APPTG-VHPT-------NQSARPNFPSIPQ 641

                                                                                   Domain VII
Cel-TRA-1 QMREVEPLQQQQQQEPMDQDLQDIRVDGDSDDEDEEEPRTPSGALLLPRGGNNGDGGFGGSG----SRASSGSGTMELSAAPISQNGSRASGSGERGMR 686
Cbr-TRA-1 HMEGAEVAPVLAPQQPLEED------DDMTDDEESGN-VAPA----VIRNGDGPDDRNSGTGSSSPRSSASSGSGTLEVAGSVA-QNGNRSSSTGERGMR 745
Cni-TRA-1 HMEVAEVAPVLAPQQPLEED------DDMSDDEESGN-VAPA----VIRNGDGPDDRNSGTGSSSPRSSASSGSGTLETAGSVA-QNGNRSSSTGERGMR 738
Cbn-TRA-1 EIETADADIDPIRVDGDDED------DDASEDEEEEARARPVGAVTAQSNGGGSDD-HSASGASSSRSSASSGSGTLEIA--PV-QNGNRSSSTGDRGMR 731
Cre-TRA-1 QIHLHNANVATA-----EED------DEMSEDDEES--LQPAQAIPASRPRDGSDDGNSGSGAGSSRSSVSSGSGTLEMAGRAT-QSESRSSGSGERGMR 727

Cel-TRA-1 SFLIADILQLAADFQNERLLSDVLDLAIFDTRDVRSLHNIYQVYIRAHKAIPI----TRRPLDWNETHQLHNLYHDPRFNRAEHQDSPAIRDRDTRFWRT 782
Cbr-TRA-1 SFLIADILQMAHDFQNGRIITDALHKAIFDTSDVTAMFHIYRTFECVCEYLQFTFSAGERLPTWPEVRTIHEYYHS-DYDRTRFHASETVRRRDNLFWRF 844
Cni-TRA-1 SFLIADILQMAHDFQNGRIITDALHKAIFDTSDDIAMFQIHRTFQCVCDYLQFTFSDGERLPTWAEVLTIHKYYHNSDYDRTRFHASETVRRRDNLFWRF 838
Cbn-TRA-1 SFLIADILQLACDFKNDRLLTDALDLAVFGTPDLKTLHKIYQLYERISH--EF-VSPSRRFISWQEVNLLHQYYHHPRFNRAGYHESPLTRTREQLFWRV 828
Cre-TRA-1 SFLIADILQLASEFGNDRIISDALNLSIFETRDINTVWHIYSLFDNACSRMDH-FETGKRLPTWQEVRVLHSYYHSPHYNRNLFHDSPAARTRETLFWRA 826

                                                                                   Domain VIII
Cel-TRA-1 IAEANTMRQRQIEPVPLD-DDDEGYFDEMVHRVQNGR----LNEQFMEGFE----SDDDDGF------EDEDDVPGLG-----IAVYRGRRRVRREALKQ 862
Cbr-TRA-1 VNYCNSDLPIGGRPI-LSVSYDEGFEESIDQGSNFGT----IPQQLISRMAAEAFDDEDDGF------DDAFDIPGLRLGGVEVLIQQQRHRVRRAALKQ 933
Cni-TRA-1 VNYCNSDLPIGGRPI-LSVSYDEGFEESIDQASDFGT----IPQQLISRMAADVSDDEDDGF------DDAFDIPGLRLGGVEVLIQQQRHRVRRAALKQ 927
Cbn-TRA-1 VNWVNLNSTRQIQPVSLDRDDDEGYFDEMASMMNHAH--VNVNNHFADEMV-------DDGFDVSDIEDDDEDVVGFG-GDIGVAIHRRRQVRHQALKQ 918
Cre-TRA-1 INSANSRREHQIQSISLNSEVDEGFEEYALRAARDGRRATSNNQSAAVRVGAQESEDEDDGFG-----DSDDDLPGLGLGGLDVIVRRRRIVRRQALKQ 921

Cel-TRA-1 ANLDIQ---EAETAGRNVGGFGDEEDRNNRGHDQDRSFLD--HYYPPMVVVVETESPQIVRDQEMMRQFEEAK----------------KNVETDEIKKR 941
Cbr-TRA-1 AQIDYP--TAEEDLGYVGGGFGQEDDD---HHSYA-IYEQ--QKTVAFVDLSEKPKVEVLTDEDFMEQFKAAEARCQARLAIEPMVKVARTVINGKVVEY 1025
Cni-TRA-1 AHIDYP--MAEEDLGYVGGGFGQEDDD---HHSYA-IYEQ--QKTVAFVDLSEKPKVEVLTDEVFMEQFKAAEARCQARLATEPMVKVARTVINGKVVEY 1019
Cbn-TRA-1 AYIDFD---ADEGFGNADGGFGAEGDGDG-HRNGD-PYLNDEARTCRLNIEMNVEEEKCASMEEFLKKHEEARARYDAYKEKQVELIMNRSIDDKELKMP 1013
Cre-TRA-1 AYMEIDESNTDNQFGVVGGGFGGENNENGDDRSYD-SFVH----TPRIDD---------SDINFIKLFKRAESDLAEKAAKLPKITEMVPIDQVQTSQQ 1006

                                                  Domain IX
Cel-TRA-1 AEAMQFGTSS----SHHHTKTLLIQRALFDKTSSVRRSLLQFITISVDQEELRQSCHATSAPQGAHVVHNVVDEFDSIMRAQEDSNNRILLSLDIPAPSA 1037
Cbr-TRA-1 TESQLFSPPV---------SRRTIYS-MLKQFPEHQENLLKFITLHVDKCELENVIPS--------------REYMSDMESS-------FFAHHRIVRAA 1094
Cni-TRA-1 TESQLSSPPV---------SRRTIYS-MLNQFPEHQENLLKFITLHVDKCELENVIPS--------------REYMSDMESS-------FFAHHRIVRAA 1088
Cbn-TRA-1 VGNGIPCDPVPAAESSTSSRRSSVSR-MLVEEPATRSTLLRFVTIHVDREEIRNQIPS--------------LEYVETPQSEEDS----LEAQHRIAVEA 1094
Cre-TRA-1 IQPEPINPPA---ACRSSTTPFSVHR-LLVEEPEFRQSLLRFVTIHVDNDEIKNNVSS--------------DEFYKLPSDE-----------RRMIEEA 1077

Cel-TRA-1 VTGVSGSITHAD-----NSALQLQQEQPTSSFSSWFPE-DDPIYALPPPP-PPPAPPRRRRSADNKDDSENIPKKPRHQF 1110
Cbr-TRA-1 LRAR-GEITEAEEAEYQQSSNLMYNQDFA---ALMFPELDESHDDFLNDL---PISSRRRSS--DSDSKEAPSMKKPRSF 1165
Cni-TRA-1 LRAR-GEITEAEEAEYQQSSNLMYNQDLQDLAALMFPE-DAPHDEFLNTP---SISSRRRSS--DSDQDGAPSMKKPRSF 1161
Cbn-TRA-1 LAGF----------------SRMTLNDRQS--ETLTPE-DEPVAVNPDDA---FNDSRRRHY---TSRDNNVPEKKSRDL 1149
Cre-TRA-1 MSAM-DGMESAE-----AAQNQAVIEEPQ---ESLFAE-DQESSSNVPSDYSIPSNPRRRSS--DSEHSEVVPEKKQRKH 1145
```

Figure S3. Alignment of five TRA-1 proteins. Domains are described in Fig. 2. The alignment was made with Muscle (https://www.ebi.ac.uk/jdispatcher/msa/muscle) and colored black for 100% identity and gray for 100% similarity among these groups of amino acids (GAVLI, FYW, CM, ST, KRH, DENQ,P) using (https://www.bioinformatics.org/sms2/color_align_cons.html).

Table S1. New mutations. The start of each wildtype sequence in Wormbase is presented in Column 2. Changes are highlighted in red, insertions in blue, and deletions as "--".

Cbr wt	*-5837499*	cgaggtc**c**gaacaa
v46		cgaggtc**T**gaacaa
Cbr wt	*-5939057*	aaat**c**ttctgaag
v48		aaat**T**ttctgaag
Cbr wt	*-5850205*	gctcaacgccgt**ggaagtt**tctcgaagaagaatggtaacggat
v182		gctcaacgccgt-------tctcgaagaagaatggtaacggat
Cbr wt	*-5837803*	cgagctcgcctcg**ttctagtg**cttccagtggaagtggc
v197		cgagctcgcctcg--------cttccagtggaagtggc
Cbr wt	*-5836789*	ttctccaccg**g**t-------ttcac
v406		ttctccaccg**C**t**CTTTTCT**ttcac
Cbr wt	*-5837286*	tccgcagcaactcatctctaga**tccgcagcaactcatctctagaatggctgctgaagcc**<br>**ttcgacgatgaagatgatggattcgacgacgcatttgacatccctggtctcagactcgg**<br>**aggtgtcgaggtcctaatccag**cagcaacgtcacagagttcgccg
v510		tccgcagcaactcatctctaga**TAGTAATGATAATGATAGAA**cagcaacgtcacagagt
Cbr wt	*-5837817*	cagtggaac**aggatcgagctcgcctcgttctagtgct**tc**cag**tggaagtggc
v511		cagtggaac----------------------------tc---tggaagtggc
Cbr wt	*-5836670*	ttccttc**tcg**tgagtatatgtctgaca
v525		ttccttc---tgagtatatgtctgaca
Cbr wt	*-5836668*	ct**c**g**tgagtatatgtctgacatggagagcagcttcttcgctcatcatcgcattg**ttcgt
v529		ct**T**g--ttcgt
Cbr wt	*-5836666*	ttctcgtga--------gtatatgtct
v530		ttctcgtga**GTACGTGA**gtatatgtct
Cbr wt	*-5836678*	gaatgtaa**ttccttctcg**tgagtatatgtctgaca
v531		Gaatgtaa----------tgagtatatgtctgaca
Cbr wt	*-5836628*	ttgagaatg--<br>-----------------aattccttc**t**cg**t**ga**g**ta**t**atgtctgacat
v532		ttgagaatg**TAATTGAGAATGTAAATCATTCTGCCTGGTTTTATAGATCAGAATCAAGC**<br>**ATTGTCCTTGAGAATGT**aattccttc**C**cg**G**ga**A**ta**C**atgtctgacat
Cbr wt	*-5840787*	atagcttggttcccgaagcagcgcagcccctccaa------------------------<br>----------------acagtggctgaatcttcggaaccaactgcagcaag
v455<br>ollas		atagcttggttcccgaagcagcgcagcccctccaa**TCTGGATTCGCGAACGAGCTTGGG**<br>**CCCGTCTTATGGAAAG**acagtggctgaatcttcggaaccaactgcagcaag
Cel wt	*-11173093*	gtgcgtcgaagt**c**ttctccaattcatcacaatctctgtt
v416		gtgcgtcgaagt**T**ttctccaattcatcacaatctctgtt
Cel wt	*-11193102*	gccaagcaaatgggttcggaggacaaacaa-----------------------------<br>------------cctggtggtggcgacgtgaaaaccgaaaatg
v472<br>ollas		gccaagcaaatgggttcggaggacaaacaa**TCTGGATTCGCGAACGAGCTTGGGCCCCG**<br>**TCTTATGGAAAG**cctggtggtggcgacgtgaaaaccgaaaatg
